# Screening Metal–Organic Frameworks for Separation of Binary Solvent Mixtures by Compact NMR Relaxometry

**DOI:** 10.3390/molecules26123481

**Published:** 2021-06-08

**Authors:** Marc Wagemann, Natalia Radzik, Artur Krzyżak, Alina Adams

**Affiliations:** 1 Institut für Technische und Makromolekulare Chemie, RWTH Aachen University, Templergraben 55, 52056 Aachen, Germany; MaWa0307@web.de (M.W.); natalieradzik@gmail.com (N.R.); 2 Department of Fossil Fuels, AGH University of Science and Technology, al. Mickiewicza 30, 30-059 Krakow, Poland; akrzyzak@agh.edu.pl

**Keywords:** MOF, separation, binary mixture, low-field NMR relaxometry

## Abstract

Metal–organic frameworks (MOFs) have great potential as an efficient alternative to current separation and purification procedures of a large variety of solvent mixtures—a critical process in many applications. Due to the huge number of existing MOFs, it is of key importance to identify high-throughput analytical tools, which can be used for their screening and performance ranking. In this context, the present work introduces a simple, fast, and inexpensive approach by compact low-field proton nuclear magnetic resonance (NMR) relaxometry to investigate the efficiency of MOF materials for the separation of a binary solvent mixture. The mass proportions of two solvents within a particular solvent mixture can be quantified before and after separation with the help of a *priori* established correlation curves relating the effective transverse relaxation times *T*_2eff_ and the mass proportions of the two solvents. The new method is applied to test the separation efficiency of powdered UiO-66(Zr) for various solvent mixtures, including linear and cyclic alkanes and benzene derivate, under static conditions at room temperature. Its reliability is demonstrated by comparison with results from ^1^H liquid-state NMR spectroscopy.

## 1. Introduction

Metal–organic frameworks (MOFs) are a class of advanced hybrid porous crystalline solids on which metal containing units and organic linkers are held together by strong chemical bonds [1,2,3,4,5]. They have gained increasing interest in recent years as an alternative to traditional nanoporous materials due to their unique combination of high porosities, large surface areas, different pore sizes, and variable topologies tailored through the use of different types of linker and metal nodes [1,2,3,4,5]. Among a large variety of possible applications [6,7,8,9,10,11,12,13,14], MOFs show great promise for separating and purifying solvent mixtures, processes of key importance in many applications [15,16,17,18,19]. It is expected that they could open an efficient alternative to existing separation procedures, which are expensive, time consuming, and very energy demanding [20,21]. As an example, distillation alone, the workhorse of chemical process industries, is responsible for 10–15% of the world’s energy consumption and contributes significantly to the greenhouse gases emissions [20,21,22]. Therefore, the improvement of current adsorption-based separation methods through the implementation of more efficient adsorbent materials is of growing interest and of fundamental importance to a competitive and environmental friendly technology development. 

The separation and purification efficiency of MOFs towards a certain solvent mixture is significantly altered by a variety of factors. They include the nature of the linker and the type of metal which can be chosen to specifically induce a preferential interaction with a certain solvent, the flexibility of the framework, a remarkable property of the MOF materials, as well as the pore size and shape which can be tuned towards size selective sieving [15,16,17,18,19,23,24,25]. A further factor, which can affect the separation efficiency of certain MOFs, is the solvent-induced breathing process [26,27,28]. Due to this effect, the porosity of the MOF increases upon solvent adsorption compared to that of the dry MOF such that the wet MOF enables the adsorption of larger molecules than the dry MOF. Up to now, tens of thousands of different MOFs have been reported and their number is strongly increasing each year [29]. Among them, there are probably several MOFs which show acceptable separation properties for a particular mixture and they are just waiting to be identified.

The efficiency of a particular MOF for the separation of solvents from binary mixtures is in many cases predicted by computational screening, currently as one of the most important tools for the fast screening of existing and hypothetical MOFs towards a particular application [30,31,32]. Another approach relies on predicting the behavior of a mixture in the presence of a MOF by taking into account the behavior of the individual solvents [33,34]. The outcomes of both approaches need however to be experimentally confirmed in the view of contradictory results reported for various solvent/MOF systems [34,35].

Transient breakthrough experiments and chromatography are the most used experimental analytical tools to investigate the performance of MOFs for mixtures separation [15,16,19]. However, they need to be combined with simulations or with other analytical tools, which are sensitive towards chemical structure to enable the identification and quantification of the individual components in the eluted mixture [15,16,19]. All these procedures are time consuming and require expensive and sophisticated equipment and trained operators. However, in order to reliably screen the huge number of existing MOFs materials, it is of key importance to identify reliable high-throughput analytical methods, which can overcome the restrictions of the existing approaches. 

Among existing analytical technics, nuclear magnetic resonance (NMR) has strongly advanced our understanding about the MOF materials in terms of structure, dynamics of the linker, and motion and diffusion of adsorbed molecules by taking advantage of dedicated high-field NMR devices and sophisticated experimental techniques [36,37,38,39,40,41,42,43]. Yet, useful microscopic information can also be gained with the help of less expensive compact low-field NMR sensors with open and closed magnet geometries. Such sensors are well suited to interrogate the behavior of protonated materials and have been already successfully applied for a detailed characterization of various materials [36,37,38,39,40]. However, their application in the context of MOFs is still relatively scarce with existing studies focusing on the relaxation and diffusion behavior of gases and solvents in MOFs [41,42,43].

In this context, we introduce compact proton NMR relaxometry with an open NMR sensor as a new tool to test the potential of a certain MOF towards the separation of solvents within a binary mixture. Compact NMR sensors are based on permanent magnet technologies [36,37]. For open compact NMR sensors, with the Profile NMR-MOUSE being the most used, the permanent magnets are arranged in well-defined positions with respect to each other to generate a thin flat slice at a fixed distance above the surface of the sensor. The acquired NMR signal steams from this slice and thus the method enables a truly non-destructive analysis of samples of different sizes and shapes by simply placing them nearby the sensor. The sensors work in strongly inhomogeneous magnetic fields, which hamper the conduction of spectroscopy measurements but are well suited for relaxation and diffusion measurements [36,37,38]. The diffusion measurements are possible because the NMR sensors with open geometries have a strong intrinsic gradient, which is in many cases around 10 to 20 T m^−1^. One of the most used measuring methods with compact NMR is, however, the CPMG (Carr, Purcell, Meiboom, Gill) pulse sequence [44,45] which allows acquiring the whole transverse relaxation decay curve within a single shot. The analysis of the CPMG decay curves is done with the help of exponential fit functions or the use of inverse Laplace transform (ILT) that delivers the effective spin–spin relaxation times *T*_2eff_.

Our method quantifies the solvent mixture composition in terms of mass proportions before and after separation by a MOF using prior established correlation curves between the transverse relaxation times *T*_2eff_ and the known mass proportions of a particular mixture. This new approach is tested by investigating the efficiency of UiO-66(Zr) in separating various binary mixtures and by comparison with the results obtained from ^1^H liquid-state NMR on exactly same samples. The proposed methodology requires minimal amount of MOF, it is simple to conduct, and once the correlation curves are established for a certain binary solvent mixture, the separation efficiency of a MOF can be determined within few minutes of measuring time. Moreover, the relaxation measurements for various solvent mixtures can be implemented for conduction in an automatic way enabling thus a high-throughput screening.

## 2. Results and Discussion

**Figure 1:**
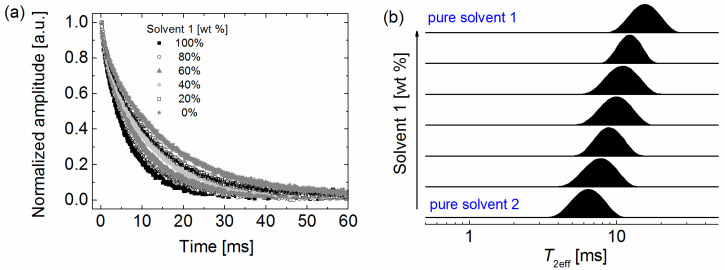
(**a**) Typical ^1^H CPMG NMR decays of two pure solvents and their mixtures at different mass ratios. The CPMG decays were recorded at room temperature. (**b**) The corresponding ILTs of the CPMG decays depicted in (**a**). The amplitudes are normalized to unity.

Typical ^1^H CPMG relaxation decays of the pure solvents and their mixtures, acquired at room temperature, along with the corresponding inverse Laplace transform (ILT) analysis are exemplarily depicted in Figure 1 and Figure S1. The extracted values of effective spin–spin relaxation times *T*_2eff_ for the pure solvents are reported in Table S1 along with literature values for the corresponding viscosity and self-diffusion coefficients. The *T*_2eff_ generally increase with increasing number of carbons and are higher for the cyclic alkane than the linear one. The lower *T*_2eff_ relaxation times for n-octane compared to cyclooctane are in agreement with reported spin–spin relaxation times *T*_2_ data obtained in a much more homogeneous magnetic field [46]. Yet, the detected trends for the other solvents are contrary to the expected behavior due to the increase in the viscosity and the reported trends in literature for the relaxation times of alkanes for which has been shown that the *T*_2_ relaxation times decrease with increasing the number of carbons [46,47]. Generally, the higher the viscosity, the shorter are the values of the *T*_2_ relaxation times and the same trend can be usually observed also for the *T*_2eff_ given that the echo-time used for the CPMG measurement is low enough to minimize the effect of self-diffusion on the CPMG decay [37,38,46,47]. The observed trend for our data can thus be explained by the impact of self-diffusion on the CPMG decays acquired using an echo-time of 70 μs in the presence of a strong static magnetic field gradient of around 20 T m^−1^. In this case, the higher the self-diffusion coefficient, the stronger is the impact of the static magnetic field gradient on the dephasing of the transverse magnetization and thus the lower the corresponding measured *T*_2eff_. A further effect comes from the used recycle delay of 4 s, which is for some of the investigated solvents of the same order as the corresponding *T*_1_ value. Nevertheless, both effects pose no impediment on discriminating the various solvents based on their *T*_2eff_ values. They can be even used to induce a higher relaxation contrast between the solvents to be investigated, another key advantage of the proposed methodology.

The ^1^H CPMG relaxation decays of the investigated binary solvent mixtures and the corresponding relaxation spectra as obtained by ILT are sensitive to the mass proportion of the two solvents within the mixture, as exemplarily shown by the results depicted in Figure 1. Single relaxation peaks are observed for the mixtures at positions in between those of the pure solvents (Figure 1b). Similar results were obtained for all investigated mixtures (Figure S1, in the Supplementary Materials). The ILT results enable the extraction of an averaged *T*_2eff_ for a particular solvent mixture as an efficient parameter for further use.

**Figure 2:**
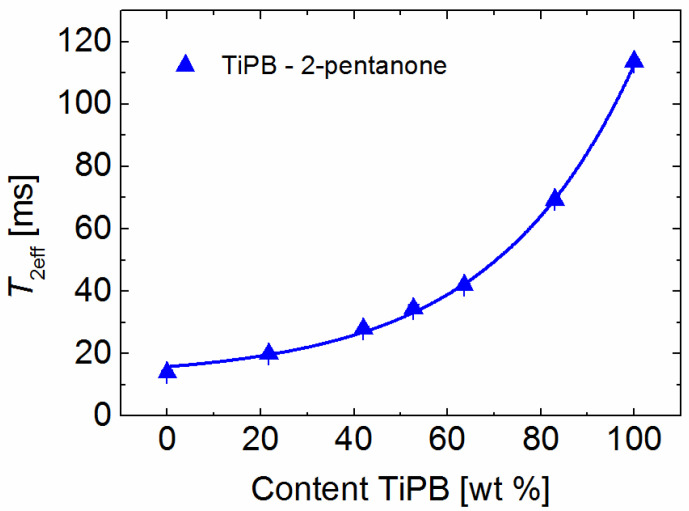
Correlation of the relaxation times with the solvent content in a binary mixture as exemplarily shown for TiPB/2-pentanone. The line depicts the fit result using the equation *T*_2eff_mixture_ = 3.72 × exp(content/30.3) + 12 with a correlation factor higher than 0.99. The relaxation times were measured at room temperature.

The variation of the obtained *T*_2eff_ of the mixtures with the mass content of the two solvents is exemplarily depicted in Figure 2 for a 1,3,5-triisopropylbenzene (TiPB)/2-pentanone mixture and in Figure S2 (in the Supplementary Materials) for all the other mixtures. All correlation curves could best be described by simple single exponential functions (Figure 2 and Figure S2). Once the correlation curves are established following the measurement and analysis of the CPMG decays, and the fit function for the correlation curve identified, a process which takes around 1 h for each mixture, the separation power of a particular MOF can be quantified within few minutes.

**Figure 3:**
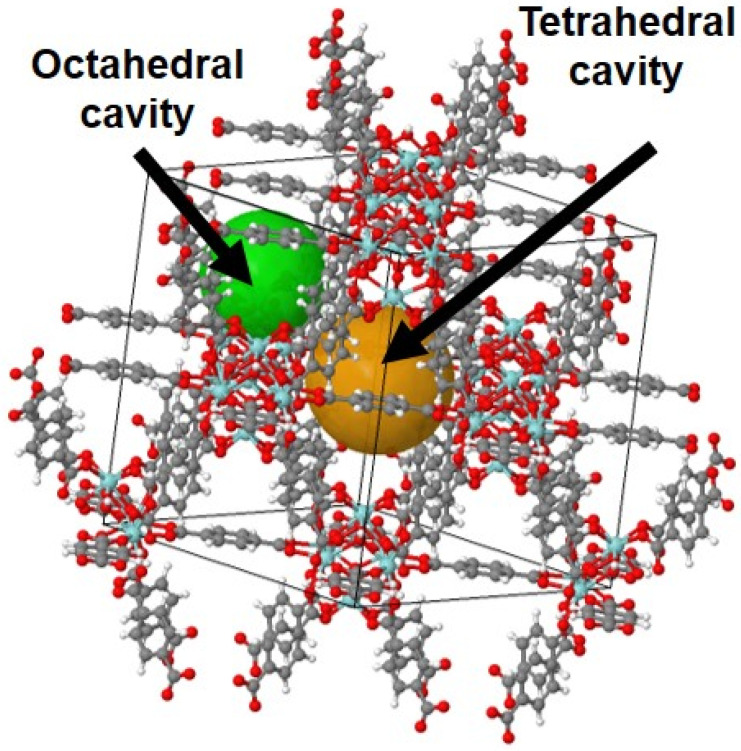
Structure of UiO-66(Zr) showing the carbon (gray), hydrogen (white), oxygen (red), zirconium (blue) and the unit cell (black outline). The tetrahedral and octahedral cavities are indicated by the green and orange spheres. Adapted from [48].

UiO-66(Zr) (Figure 3) was chosen as a model MOF to test our methodology in the view of its high thermal, mechanical, and chemical stability, well-controlled synthesis procedure, and great promise in the field of separation [23,48,49,50,51,52,53,54]. This MOF is built from Zr_6_O_6_(CO_2_)_12_ nodes connected via terephthalate linkers. Its pore system contains tetrahedral and octahedral cavities (free diameters of about 8 Å and 11 Å) [50]. Both cavities are connected by a small triangular window with a pore aperture of about 6.5 Å [50] which needs to be passed by any molecule entering the pores of UiO-66 acting thus as a sieve for larger molecules. 

**Figure 4:**
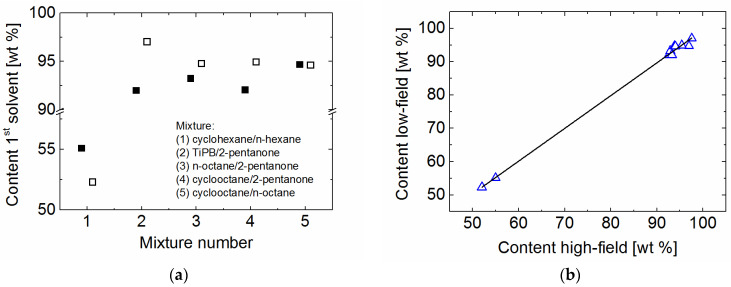
(**a**) Content of the first solvent before (filled symbols) and after (empty symbols) extraction by MOF as calculated based on the *T*_2eff_ values given in Figure S3. For the n-hexane/cyclohexane a mixture with around 55 wt % cyclohexane was tested. The errors of the obtained values are lower than 0.5%. (**b**) Correlation of the solvent content in the mixture before and after separation as determined by low-field relaxometry and high-field liquid-state spectroscopy. For the sake of comparison, the results of all mixtures presented in Figure 4a are included. The continuous line is the linear fit result with a correlation factor of 0.997.

The separation efficiency of powdered UiO-66 due to selective adsorption was tested at room temperature under static conditions for different solvent mixtures by measuring the solvent mixture following equilibration with the MOF and subsequent removal (see the experimental section for more details). The relaxation times of the binary mixtures before and after contact with UiO-66 are different (Figure S3, in the Supplementary Materials). This indicates changes in the mass proportion of the two solvents in the mixture (Figure 4a). In particular, one observes that the content of both n-octane and cyclooctane increases in the mixture with 2-pentanone after separation. This indicates that UiO-66 has a preferential adsorption for 2-pentanone compared to the other two solvents, probably due to a stronger interaction of the 2-pentanone with UiO-66. Furthermore, a partially removal of 2-pentanone from the mixture with TiPB was detected. Given that 2-pentanone should readily enter into UiO-66 and the kinetic diameter of TiPB of about 8.5 Å is much larger than the size of the UiO-66 window, a combination between a sieving mechanism and a blocking of the UiO66 windows by TiPB which prevents that 2-pentanone enters the pores can explain the observed behavior.

The proportion of n-octane and cyclooctane remains largely the same before and after the filtration indicating that the two isomers fail to be separated in the liquid phase under static conditions by powdered UiO-66(Zr). This is an unexpected result because one can assume that n-octane can easily enter into the MOF given that the kinetic diameter of the n-octane is much smaller than the size of the UiO-66 window. Monte Carlo simulations of these two solvents inside the MOF indicate that at zero coverage, n-octane is proportionally distributed between the small and the large cavities and cyclooctane can fit even inside the small, tetrahedral, cavity [52]. However, the simulations give no hint if the cyclooctane, with his kinetic diameter of 8 Å can enter the MOF through the window of 6.5 Å. Given that, to our knowledge, no reports about the separation of the n-octane and cyclooctane by UiO66(Zr) in the liquid phase at room temperature are reported, the raisons behind our observations are not clear. Yet, it has been reported that n-alkanes have the same conformation in a pure liquid state and in a gas state, but they can change conformation in the presence of other solvents [55,56]. Thus, a possible explanation for our observations would be that n-octane changes conformation in the presence of cyclooctane towards a more coil structure and with this its kinetic diameter increases making thus difficult to pass through the MOF window. A further possible explanation of the observed behavior could be related to a solvent induced-breathing process of the MOF. This would lead to an increase in the size of the window such that also cyclooctane could enter the MOF. A similar solvent induced-breathing process had to be taken into account to explain the dependence of the self-diffusion coefficients of methane with the pressure in UiO-66(Zr) [57]. Additional studies are planned for the future to elucidate if a combination of both processes is involved in the observed behavior for this mixture or is largely due only to a change in conformation. 

However, a small increase in the content of n-hexane compared to cyclohexane after separation was observed. This is consistent with the reported preferential adsorption of cyclic alkanes over the linear alkanes in UiO-66 measured using vapor phase breakthrough experiments on which the mixtures were diluted using carrier gases such as helium [51,52]. As the pore size of UiO-66 are large enough to accommodate both n-hexane and cyclohexane possible explanations of the much lower separation efficiency are a competitive co-adsorption of both components, the solvent-solvent interaction which in liquid state should play a non-negligible role as well as the particular experimental conditions [58].

**Figure 5:**
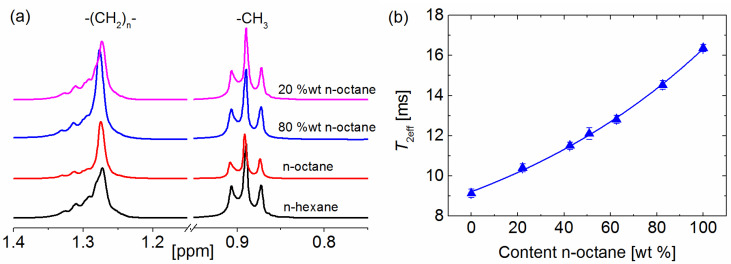
(**a**) Proton liquid-state spectra of pure n-octane, pure n-hexane, and two mixtures of them measured at room temperature. Both solvents show signals in the same range of chemical shifts. (**b**) Correlation of the low-field NMR relaxation times with the n-octane content in a binary mixture with n-hexane. The line depicts the fit result using the equation *T*_2eff_mixture_ = 5.03 × exp(content/113) + 4.17 with a correlation factor higher than 0.99.

The quantified mass proportions of all mixtures by low-field relaxometry approach show an excellent agreement with the results from liquid-state ^1^H spectroscopy. Proton NMR spectroscopy is another method which can be used to easily quantify the content of certain solvents in a mixture without the need of an a priori calibration or the use of advanced data analysis but requires more sophisticated equipment and for complicated solvent structures measurements at high-magnetic field. Furthermore, the spectra alone fail to give the needed information when applied to certain mixtures including linear alkanes as, for example, for n-hexane/n-octane (Figure 5a). The identical appearance of the spectra of the pure solvents and their mixtures prevents the quantification of the solvent content inside the mixture using solely the differences in the chemical shift of the functional groups. A differentiation between the two solvents and their mixtures can however very easily be achieved with relaxation measurements under the experimental conditions already described (Figure 5b).

The separation of a n-octane/n-hexane mixture by UiO-66 with initially 82 wt % n-octane as determined by weight was also tested. The *T*_2eff_ of this mixture of 14.5 ms translates into 81.32 wt % n-octane according to the correlation equation, in good agreement with the weighted value. No separation could be observed for this mixture by UiO-66, probably due to raisons mentioned already before for other binary mixtures.

## 3. Materials and Methods

### 3.1. Samples

All solvents investigated in this study (2-pentanone, n-hexane, cyclohexane, n-octane, cyclooctane, and 1,3,5-triisopropylbenzene (TiPB)) were purchased from Sigma-Aldrich (Germany). Different binary mixtures of the mentioned solvents (cyclooctane/n-octane, cyclooctane/2-pentanone, n-octane/2-pentanone, n-hexane/cyclohexane, and TiPB/2-pentanone) were then prepared by mixing the two solvent components at different mass proportion with a help of a syringe. Due to possible evaporation issues during the preparation of the mixture, the estimated mass proportion was controlled by ^1^H high-field liquid-state spectroscopy measurements. The binary mixtures were sealed in a glass container in order to keep the concentration constant during the NMR measurement.

The MOF UiO-66(Zr) was purchased from Strem Chemicals (USA). According to the manufacturer, this MOF has a particle size of 0.2–0.5 μm, 1000–1600 m^2^ g^−1^ BET, and a pore volume of 0.3–0.5 cm^3^ g^−1^ [59]. Following purification at 220 °C overnight under vacuum conditions, the MOF powder was stored under argon atmosphere. The activate MOF retains its crystalline structure as demonstarted by the experimental diffraction patterns (Figure S4, in the Supplementary Materials). All further handling involving MOF samples was also performed under an argon atmosphere to avoid any possible water adsorption from the atmosphere.

To investigate the applicability of proton NMR relaxometry as a fast analytical tool to test the separation power of a certain MOF material, the above mentioned mixtures of the two solvents with a volume proportion of about 1:12 (unless else stated) was used. A total of 65 µL solvent was added for 10 mg of dry MOF. This solvent amount was chosen considering the total amount of solvent that the MOF can uptake. Shortly before starting the measurements, the needed amount of MOF powder was loosely placed in a glass bottle under an argon atmosphere and at room temperature. The mixture of solvents was then gently poured on the top of the MOF using a syringe. The bottle was then immediately closed with a cap sealed with an elastic band and then covered with Teflon coating to prevent the evaporation of hydrocarbons. The prepared system was then left for about 2 h at room temperature to reach an equilibrium state. This equilibration time was confirmed by monitoring the changes in the relaxation times and the ^1^H spectra of the solvent mixture after different contact times with the metal–organic framework (Figure S5, in the Supplementary Materials). Then a filter was used to remove the MOF and the left solvent mixture was measured by NMR. Each sample was weighed before and after NMR measurements to be sure that no solvent evaporated within the frame of the measurement takes place.

### 3.2. NMR Experiments

**Figure 6:**
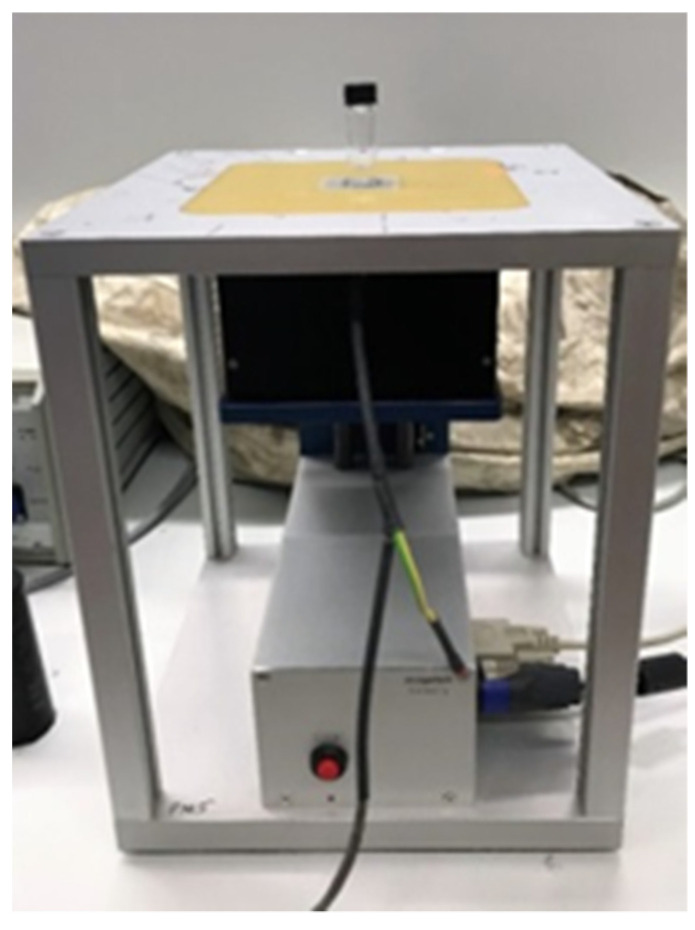
The experimental set-up used to measure the relaxation times of the pure solvents and their mixture before and after the solvent separation by a MOF (here UiO-66(Zr)). The sample to be investigated is simply placed on the top of the profile NMR-MOUSE.

The NMR experiments were performed at room temperature with a single-sided, portable NMR-MOUSE sensor having a static gradient field of about 20 T m^−1^ and working at a proton resonance frequency of 18.2 MHz (Figure 6). A Bruker minispec spectrometer (Germany) was used for pulse generation and signal acquisition. Effective ^1^H spin–spin relaxation times *T*_2eff_ of the pure solvents and their mixtures were determined at room temperature by employing a CPMG pulse sequence [44,45] with an echo time of 0.07 ms. The waiting time between two scans was set for all samples to 4 s in order to avoid heating effects during the measurements. In order to decrease the uncertainty, each measurement was performed three times. The error of the extracted *T*_2eff_ values was for all samples less than 1% (see Table S2 for typical values, in the Supplementary Materials). For understanding the trends obtained for the *T*_2eff_ values of the pure solvents, literature values, where available, are reported for the corresponding viscosities and self-diffusion coefficients [60,61,62,63].

The analysis of all CPMG decays could be best done with the help of a single exponential function for the pure solvents and the mixtures (Figure S1). The distribution of the effective transverse relaxation times was obtained by performing an inverse Laplace transform (ILT) of CPMG relaxation decays. 

Proton liquid-state high-field NMR measurements were performed at room temperature using a Bruker Ultrashield magnet operating at a proton frequency of 400 MHz and controlled by an AVANCE 3 console. The pure solvents and the various solvent mixtures were transferred to a 5 mm NMR tube hosting deuterated chloroform. The ^1^H spectra were acquired after a single 90° radio-frequency pulse with a recycle delay of ten seconds. Evaluation of the mixture ratios was done via integration of component specific peaks. The ^1^H spectra of all mixtures before and after the separation are depicted in Figures S6–S10 (in the Supplementary Materials) along with the signal assignment and the values of the integral of interest. 

## 4. Conclusions

This work introduces a simple and fast way of quantifying the separation degree of a binary solvent mixture by a MOF material with exemplification on UiO-66(Zr) using a small and low-cost single-sided NMR device. The proposed method is based on correlation curves between the proton effective transverse relaxation times *T*_2eff_ and the mass proportions of the two solvents. Once the correlation curves were established, the mass proportions in the filtered mixture can be obtained within a few minutes with great accuracy, as demonstrated by the excellent agreement with the results from liquid-state NMR spectroscopy. The proposed approach can be even applied to characterize solvent mixtures where NMR spectroscopy alone fails. It could help identifying experimental conditions that improve the separation of the mixture components by systematically investigating the impact of particular experimental parameters, such as, e.g., the separation temperature or the presence or absence of a carrier gas. The use of other MOFs with higher separation selectivity for the studied binary mixtures than UiO-66(Zr) would further highlight the potential of this novel method. The whole NMR setup can be introduced inside a synthesis laboratory and it is amenable to automation; thus, helping to save time in searching for adequate separation conditions and MOFs for a particular solvent mixture. 

## Supplementary Materials

The following are available online. Figure S1: Exemplarily ILT spectra of various investigated mixtures. Figure S2: Correlation curves for different binary mixtures. The continuous lines are the fit results using a single exponential function and has for all samples a correlation factor higher than 0.99. Figure S3: Changes in the effective relaxation times before (closed symbols) and after separation (open symbols) with UiO-66(Zr) for all investigated mixtures. Figure S4: Experimental diffraction patterns of activated UiO-66 (Zr) showing the presence of a fully crystalline structure. Figure S5: Typical ILT of the proton CPMG decays (a) and 1H spectra (b) of the solvent mixture after different mixing times with the metal–organic framework. The solvent-MOF system equilibrates within around 2 h, as no changes between the results can be detected at longer times. Figure S6: 1H high-field spectra of the binary mixture cyclohexane—n-hexane before (top) and after the separation (bottom) by UiO-66(Zr). Figure S7: 1H high-field spectra of the binary mixture TiPB—2-pentanone before (top) and after the separation (bottom) by UiO-66(Zr). The two solvents were mixed in a ratio of about 1:12 as described in the experimental section. Figure S8: 1H high-field spectra of the binary mixture n-octane—2-pentanone before (top) and after the separation (bottom) by UiO-66(Zr). The two solvents were mixed in a ratio of about 1:12 as described in the experimental section. For better view of smaller signals, the signals of n-octane are not shown in full amplitude. Figure S9: 1H high-field spectra of the binary mixture cyclooctane—pentanone before (top) and after the separation (bottom) by UiO-66(Zr). The two solvents were mixed in a ratio of about 1:12 as described in the experimental section. For better view of smaller signals, the signal of cyclooctane is not shown in full amplitude. Figure S10: 1H high-field spectra of the binary mixture cyclooctane—n-octane before (top) and after the separation (bottom) by UiO-66(Zr). The two solvents were mixed in a ratio of about 1:12 as described in the experimental section. For better view of smaller signals, the signal of cyclooctane is not shown in full amplitude. Table S1 (in the Supplementary Materials): Proton transverse relaxation times T2eff of the pure solvents measured using the NMR-MOUSE at room temperature and using the experimental conditions described in the experimental section. Diffusion coefficients and viscosity values are also shown for better understanding the obtained values for the T2eff. Table S2: Reproducibility of the NMR relaxation measurements with exemplification on different mixtures.
