# Supplementary material for: Screening Metal–Organic Frameworks for Separation of Binary Solvent Mixtures by Compact NMR Relaxometry
Source: Molecules. 2021 Jun 8;26(12):3481. doi: 10.3390/molecules26123481 (PMC8228364; doi:10.3390/molecules26123481)
Supplement: Supplementary file 1 [file molecules-26-03481-s001.zip › molecules-1232421-SI.pdf]

## Supporting Information

# Screening Metal-Organic Frameworks for Separation of Binary Solvent Mixtures by Compact NMR Relaxometry


**Marc Wagemann[1], Natalia Radzik[1], Artur Krzyżak[2], and Alina Adams[1*]**

1 Institut für Technische und Makromolekulare Chemie, RWTH Aachen University, Templergraben 55, 52056 Aachen, Germany

2 AGH University of Science and Technology, al. Mickiewicza 30, 30-059 Krakow, Poland

* Correspondence: Alina.Adams@itmc.rwth-aachen.de; Tel.: (+49)-241-8026428


## Results

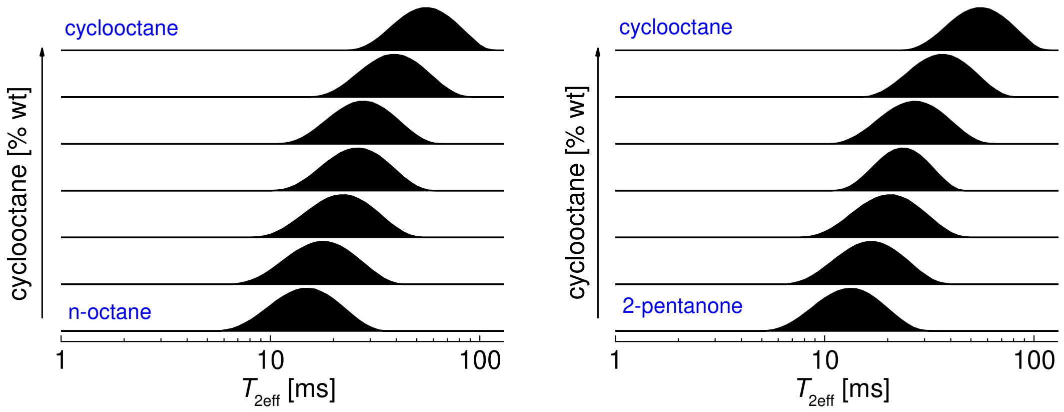

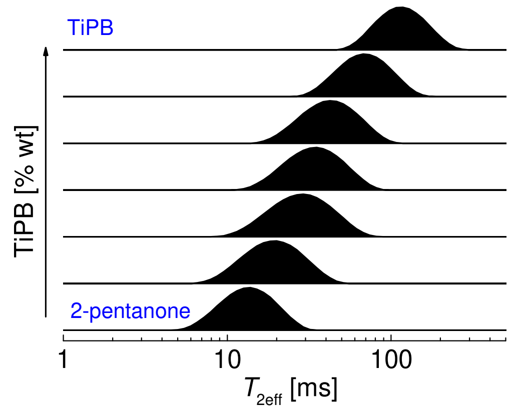


**Figure S1**. Exemplarily ILT spectra of various investigated mixtures.

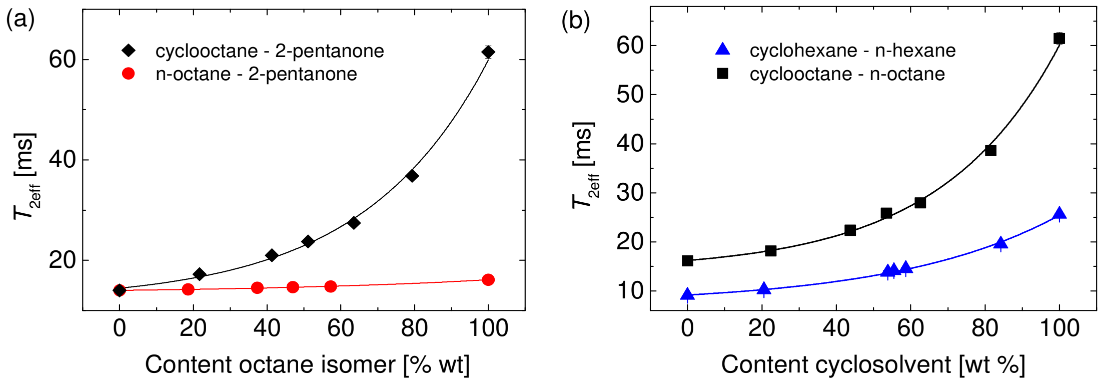


**Figure S2**. Correlation curves for different binary mixtures. The continuous lines are the fit results using a single exponential function and has for all samples a correlation factor higher than 0.99. The fit equations are: $T_{2eff}$ (cyclooctane – 2-pentanone) = 4.04*exp(content/39.53)+10.67; $T_{2eff}$ (n-octane – 2-pentanone) = 0.60*exp(content/68.03)+13.48; $T_{2eff}$ (cyclohexane – n-hexane) = 1.75*exp(content/44.76)+7.18; $T_{2eff}$ (cyclooctane – n-octane) = 1.49*exp(content/29.15)+15.15.

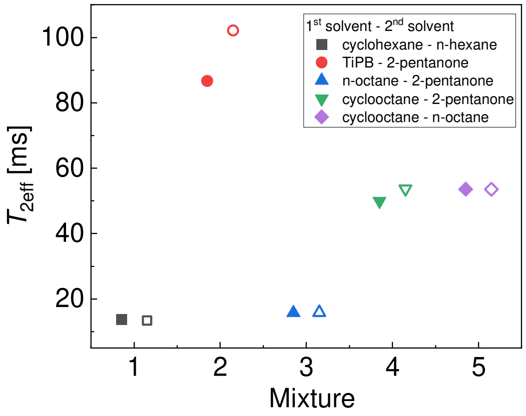


**Figure S3**. Changes in the effective relaxation times before (closed symbols) and after separation (open symbols) with UiO-66(Zr) for all investigated mixtures.

**Table S1**. Proton transverse relaxation times $T_{2eff}$ of the pure solvents measured using the NMR-MOUSE at room temperature and using the experimental conditions described in the experimental section. Diffusion coefficients and viscosity values are also shown for better understanding the obtained values for the $T_{2eff}$.

| Solvent | $T_{2eff}$ [ms] | Diffusion coefficient at room temperature [$10^{-9}$ m$^2$ s$^{-1}$] | Viscosity [mPa s] | Kinetic diameter [Å] |
|---|---|---|---|---|
| n-pentane | 6.34 | 5.72[1] | 0.24 (20 °C)[1] | 4.3[5] |
| 2-pentanone | 14.01 | - | 0.50 (20 °C)[1] | |
| n-hexane | 9.07 | 4.1[1] | 0.3 (25 °C)[2] | 4.3[5] |
| cyclohexane | 25.61 | 1.48[3] | 0.89 (25 °C)[3] | 6[5] |
| n-octane | 16.13 | 2.35[3] | 0.51 (25 °C)[3] | 4.3[5] |
| cyclooctane | 61.46 | 0.56[3] | 1.96 (25 °C)[3] | 8[6] |
| TiPB | 113.62 | 0.14[4] | 3.53 (20 °C)[4] | 8.5[7] |

[1]https://en.wikipedia.org
[2]https://wiki.anton-paar.com/en/hexane
[3]Tofts, P. S.; Lloyd, D.; Clark, C. A.; Barker, G. J.; Parker G. J. M.; McConville, P.; Baldock, C.; Pope, J. M. Test liquids for quantitative MRI measurements of self-diffusion coefficient in vivo. *Magn. Reson. Med.* **2000**, 43, 368–374.
[4]Fischer, J.; Weiss, A. Transport properties of liquids. V. self-diffusion, viscosity, and mass density of ellipsoidal shaped molecules in the pure liquid phase. *Ber. Bunsenges. Phys. Chem.* **1986**, 90, 896–905.
[5]H Funke, H. H.; Argo, A. M.; Falconer, J. L.; Noble, R. D. Separations of Cyclic, Branched, and Linear Hydrocarbon Mixtures through Silicalite Membranes. *Ind. Eng. Chem. Res.* **1997**, *36*, 137-143.
[6]Chua, L. M.; Hitchcock, I.; Fletcher, R. S.; Holt, E. M.; Lowe, J.; Rigby, S. P. Understanding the Spatial Distribution of Coke Deposition within Bimodal Micro-/Mesoporous Catalysts using a Novel Sorption Method in Combination with Pulsed-gradient Spin echo NMR. *J. Catal.* **2012**, 286, 260–265.
[7]Van der Perre, S.; Van Assche, T.; Bozbiyik, B.; Lannoeye, J.; De Vos, D. E.; Baron, G. V.; Denayer, J. F. M. Adsorptive Characterization of the ZIF-68 Metal-Organic Framework: A Complex Structure with Amphiphilic Properties. *Langmuir* **2014**, 30, 8416–8424.

## Materials and Methods

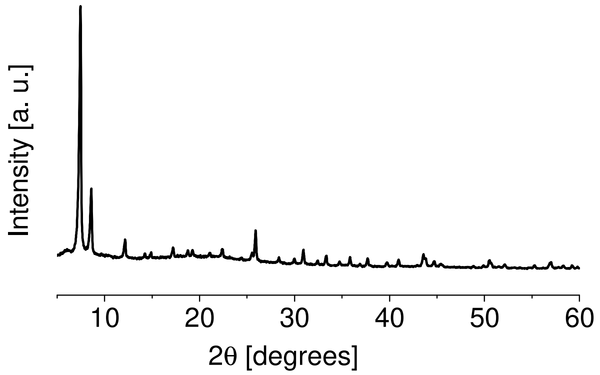


**Figure S4**. Experimental diffraction patterns of activated UiO-66 (Zr) showing the presence of a fully crystalline structure.

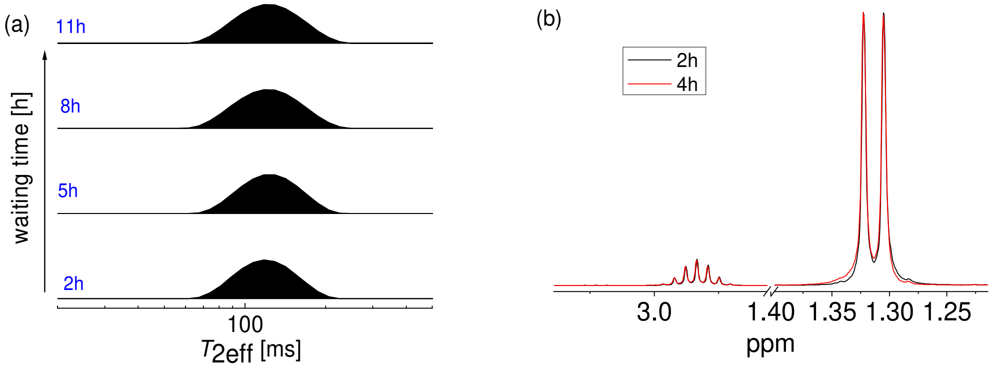


**Figure S5**. Typical ILT of the proton CPMG decays (a) and $^{1}H$ spectra (b) of the solvent mixture after different mixing times with the metal–organic framework. The solvent-MOF system equilibrates within around 2 hours as no changes between the results can be detected at longer times.

**Table S2**. Reproducibility of the NMR relaxation measurements with exemplification on different mixtures.

| **Solvent** | | **Solvent ratio [% wt]** | | **$T_{2eff}$ [ms]** | | | **Averaged $T_{2eff}$ [ms]** | **Standard deviation [ms]** |
|---|---|---|---|---|---|---|---|---|
| **1** | **2** | **1** | **2** | **Exp.1** | **Exp.2** | **Exp.3** | | |
| n-octane | 2-pentanone | 15 | 85 | 14.36 | 14.16 | 14.07 | 14.20 | 0.15 |
| cyclooctane | n-octane | 54 | 46 | 26.24 | 25.73 | 25.83 | 25.93 | 0.27 |
| cyclooctane | 2-pentanone | 75 | 25 | 37.06 | 36.94 | 36.50 | 36.83 | 0.29 |
| TiPB | 2-pentanone | 23 | 77 | 29.17 | 28.92 | 28.74 | 28.94 | 0.22 |

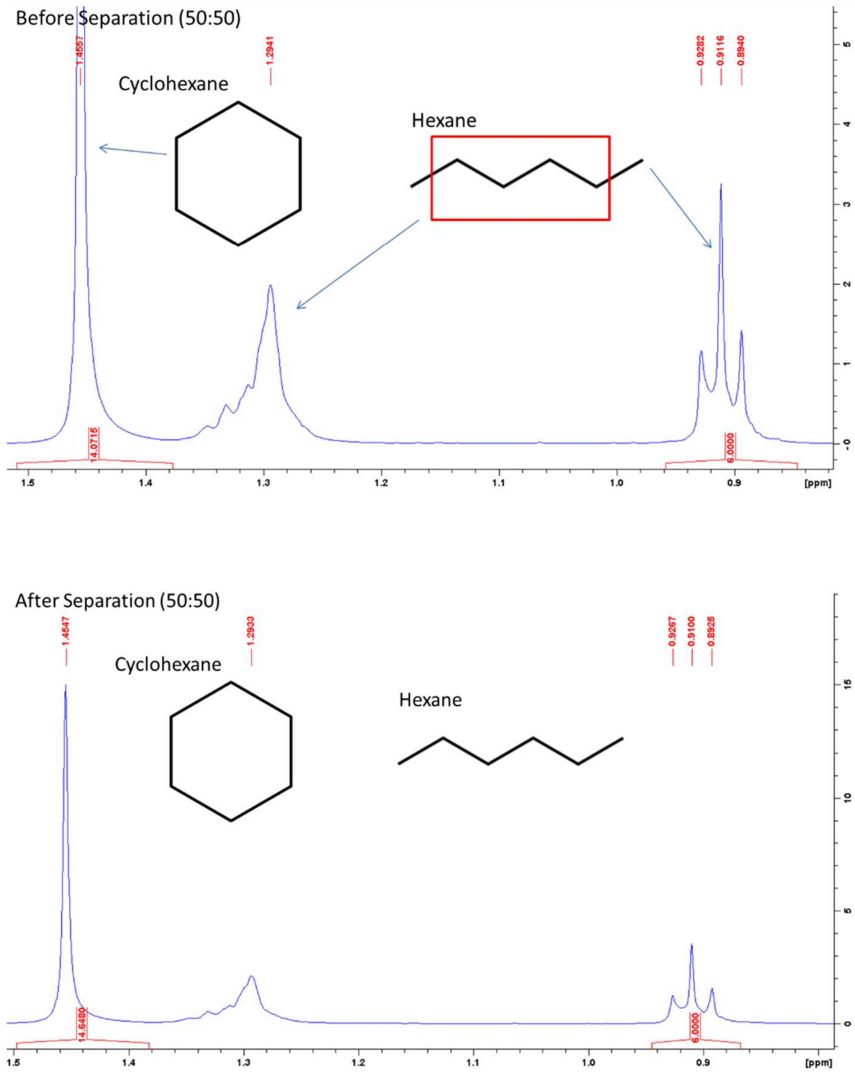


**Figure S6.** $^1$H high-field spectra of the binary mixture cyclohexane – n-hexane before (top) and after the separation (bottom) by UiO-66(Zr). The two solvents were mixed in a ratio of about 50:50.

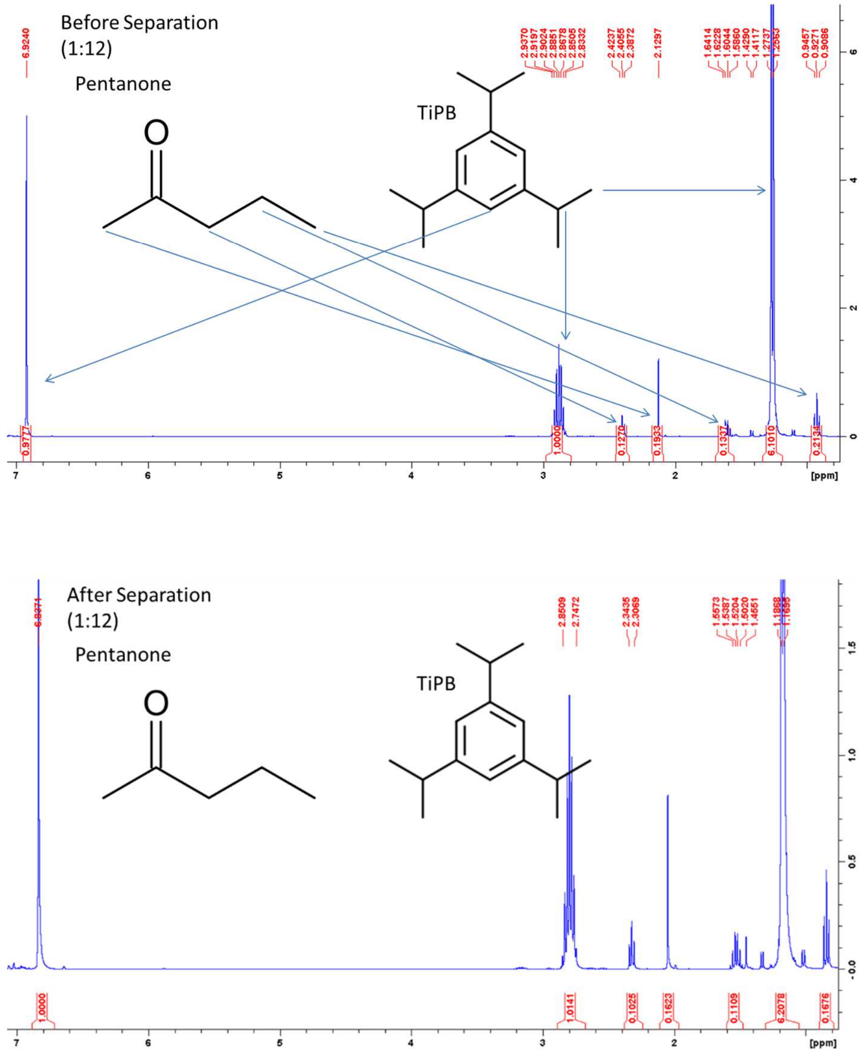


**Figure S7.** $^{1}$H high-field spectra of the binary mixture TiPB – 2-pentanone before (top) and after the separation (bottom) by UiO-66(Zr). The two solvents were mixed in a ratio of about 1:12 as described in the experimental section.

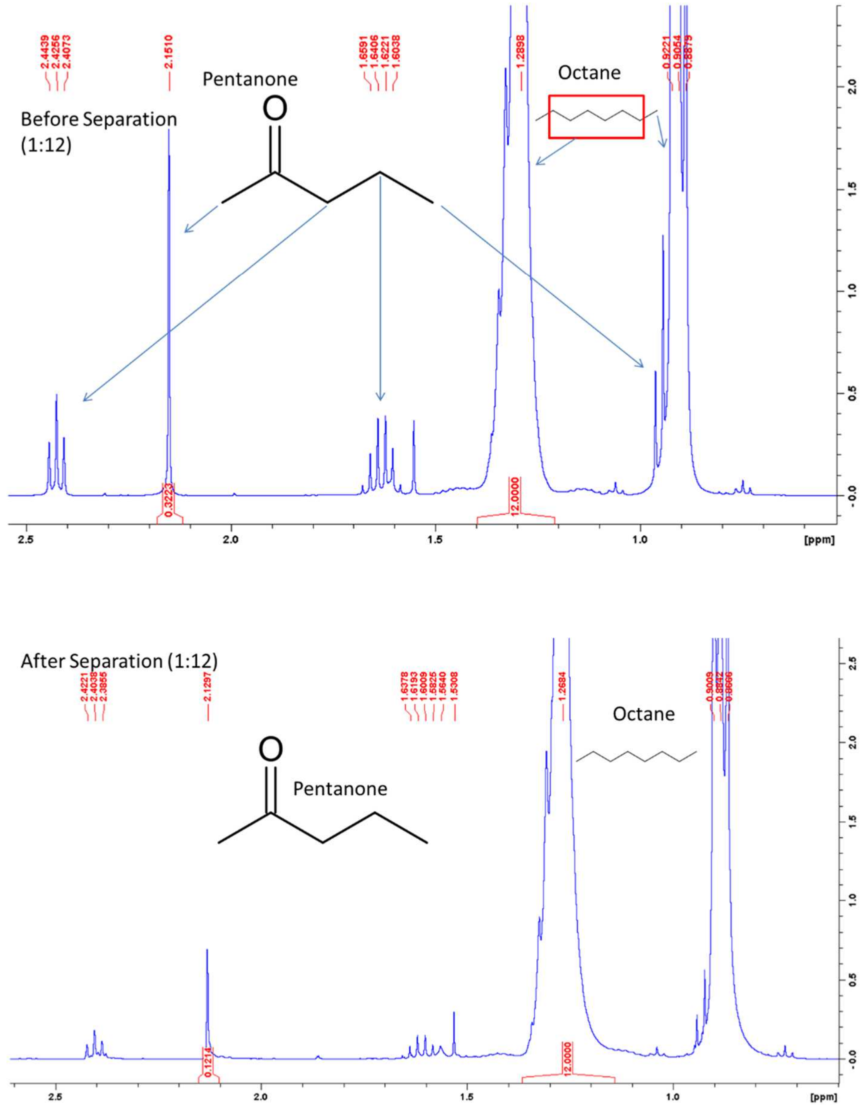


**Figure S8.** $^{1}$H high-field spectra of the binary mixture n-octane – 2-pentanone before (top) and after the separation (bottom) by UiO-66(Zr). The two solvents were mixed in a ratio of about 1:12 as described in the experimental section. For better view of smaller signals, the signals of n-octane are not shown in full amplitude.

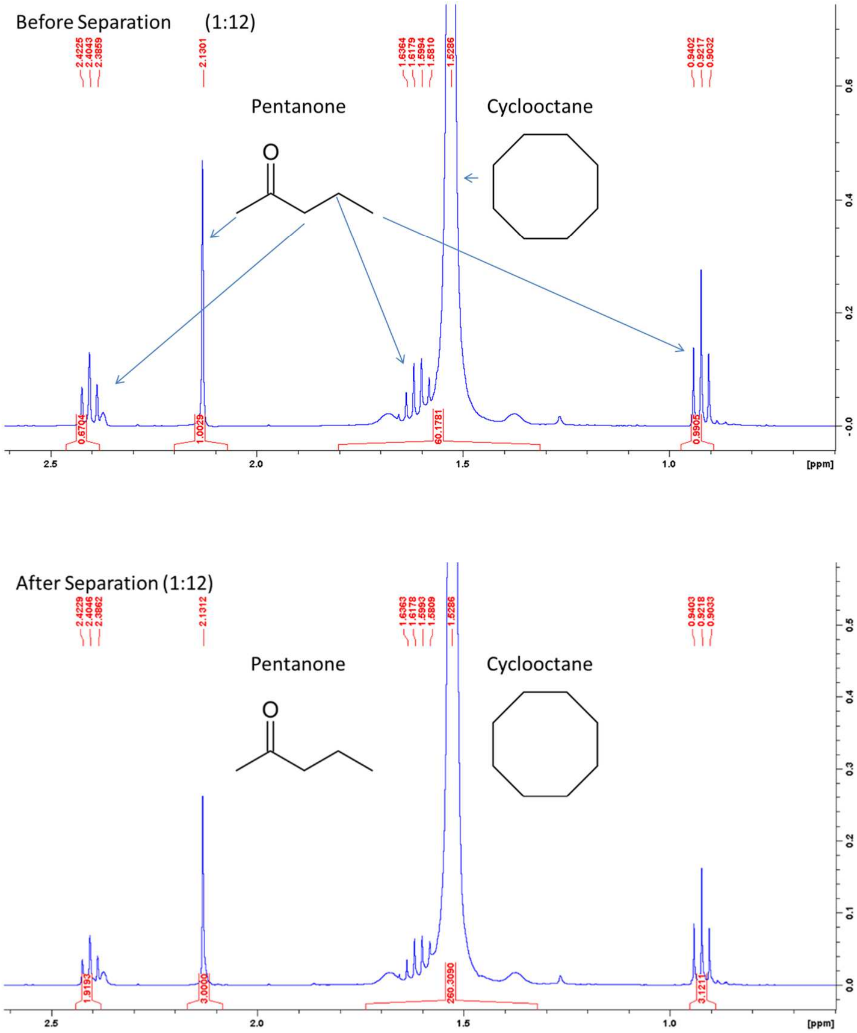


**Figure S9.** $^{1}H$ high-field spectra of the binary mixture cyclooctane – pentanone before (top) and after the separation (bottom) by UiO-66(Zr). The two solvents were mixed in a ratio of about 1:12 as described in the experimental section. For better view of smaller signals, the signal of cyclooctane is not shown in full amplitude.

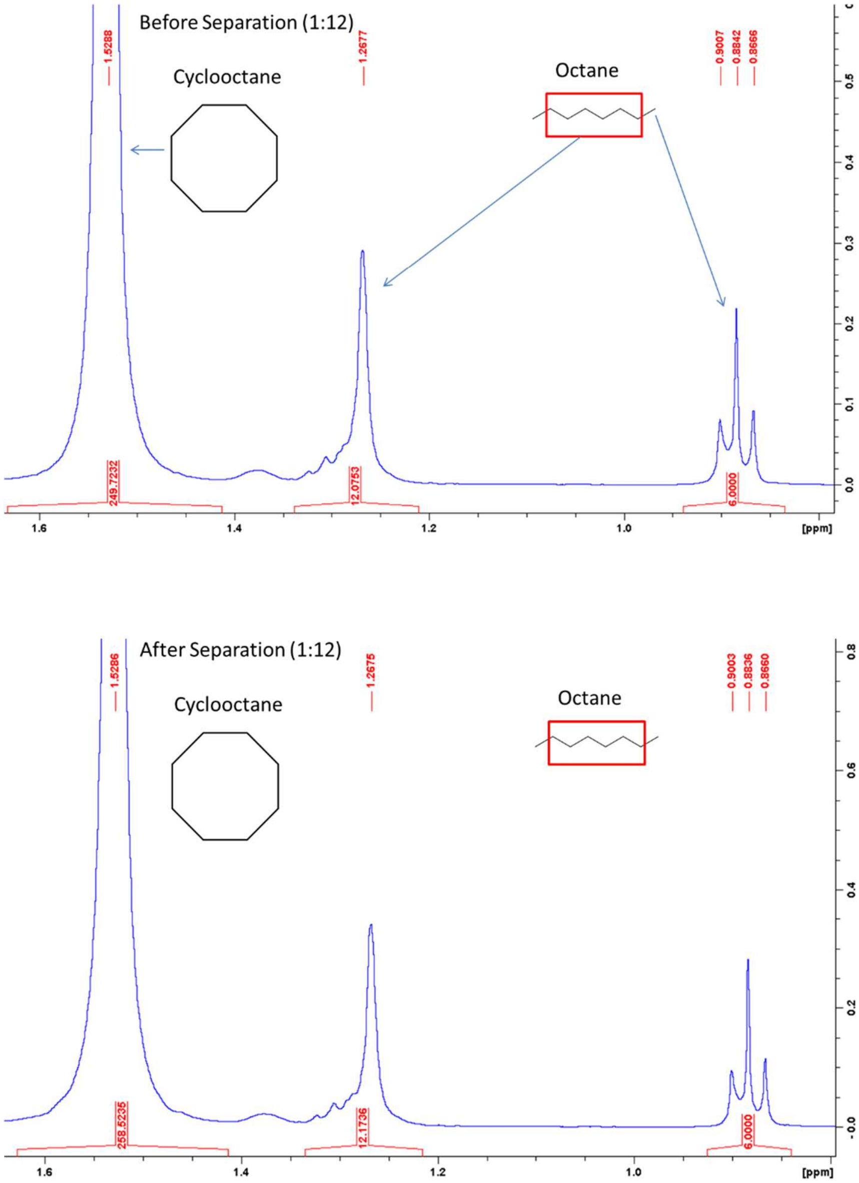


**Figure S10.** $^1H$ high-field spectra of the binary mixture cyclooctane – n-octane before (top) and after the separation (bottom) by UiO-66(Zr). The two solvents were mixed in a ratio of about 1:12 as described in the experimental section. For better view of smaller signals, the signal of cyclooctane is not shown in full amplitude.
